# Supplementary material for: Identification of MicroRNAs Associated with Prediabetic Status in Obese Women
Source: Int J Mol Sci. 2023 Oct 27;24(21):15673. doi: 10.3390/ijms242115673 (PMC10648886; doi:10.3390/ijms242115673)
Supplement: Supplementary file 1 [file ijms-24-15673-s001.zip › ijms-2645076-supplementary.pdf]

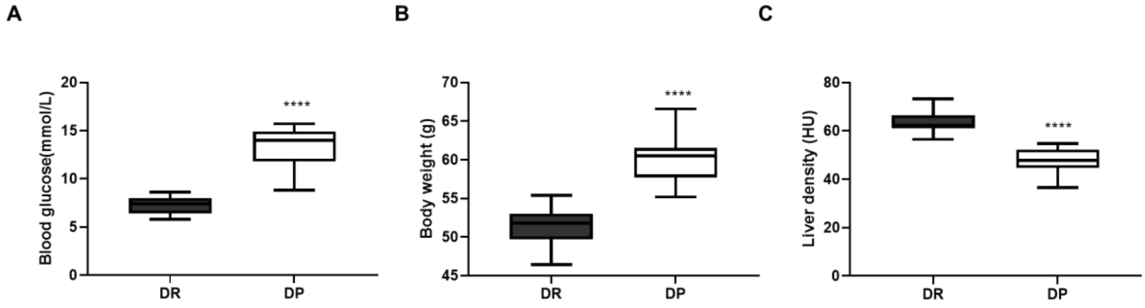

Figure S1. Phenotypic data from DR and DP mice. (A) Blood glucose levels (B) body weight (C) liver density in diabetes-prone and resistant mice. Liver density was evaluated by computed tomography (CT) at indicated time points. Higher Hounsfield units (HU) reflect lower hepatic fat content. N=15 per group. Data are mean ± SEM. Differences between the groups were calculated by welch t.test ***P < 0.001.

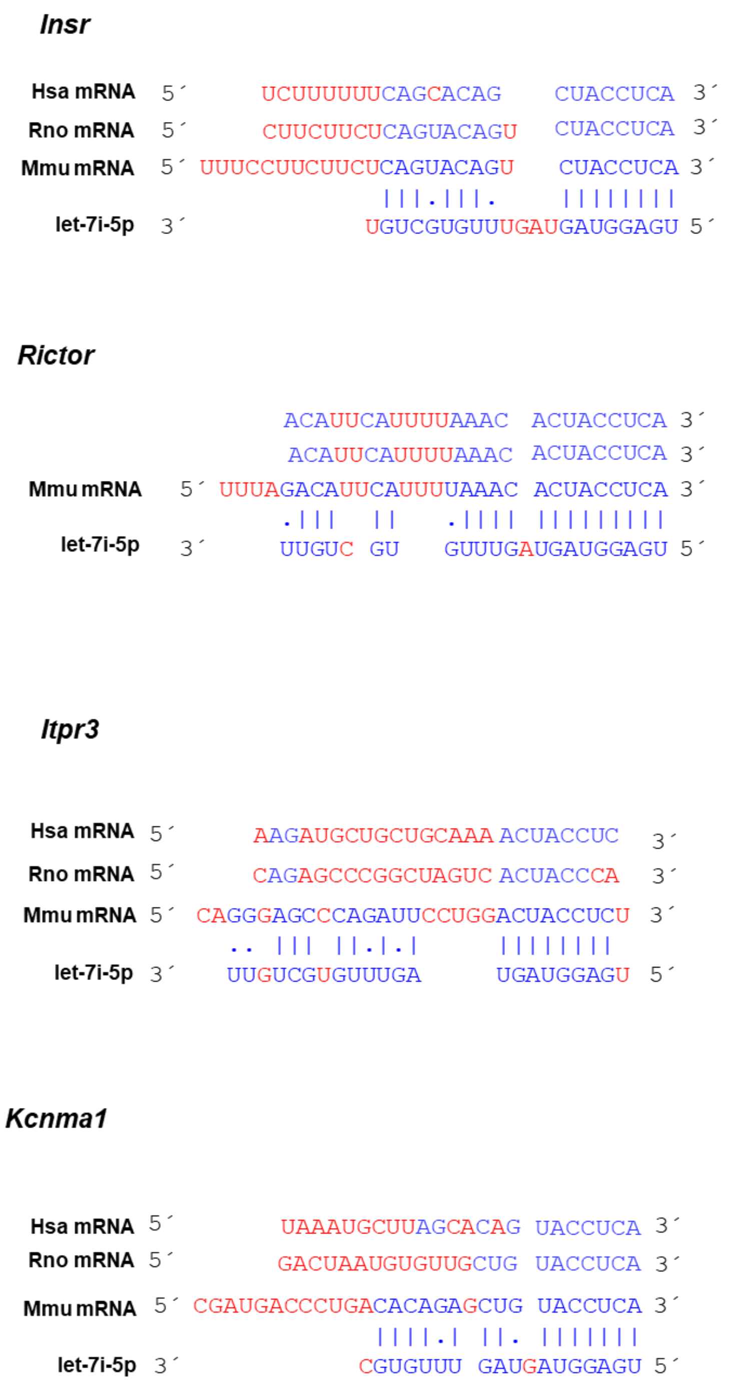

Figure S2. Let-7i-5p seed sequence alignment. Examples of Alignment of let-7i-5p seed sequence with the target sequences of selected candidates from insulin signaling pathway in mouse (Mmu), rat (Rno) and human (Hsa). The alignment of mouse sequences was done with DIANA Tools, while rat and human mRNA sequences were extracted from Ensembl.org. The complementarity between mRNA and miRNA sequences is shown in blue and non-complementary sequences in red.

Supplementary Table S1: Generalized linear models

hsa-miR-652-3p

Parameter	Coefficient	95% CI	t(60)	p	Std. Coef.	Std. Coef. 95% CI	Fit
(Intercept)	1.39	[0.78, 2.00]	4.44	< .001	0.17	[-0.11, 0.46]	
AGE	-4.22E-03	[-0.01, 0.00]	-1.4	0.162	-0.18	[-0.42, 0.07]	
BMI	6.57E-04	[-0.01, 0.02]	0.09	0.93	0.01	[-0.24, 0.26]	
Group [IFG]	-0.15	[-0.30, -0.01]	-2.12	0.034	-0.56	[-1.07, -0.04]	
AIC							19.69
BIC							30.48
R2							0.1
Sigma							0.27

hsa-miR-130a-3p

Parameter	Coefficient	95% CI	t(60)	p	Std. Coef.	Std. Coef. 95% CI	Fit
(Intercept)	0.77	[-0.37, 1.91]	1.32	0.19	0.15	[-0.14, 0.44]	
AGE	0.00	[-0.01, 0.01]	-0.01	1.00	0.00	[-0.25, 0.25]	
BMI	0.01	[-0.02, 0.04]	0.76	0.45	0.10	[-0.15, 0.35]	
Group [IFG]	-0.24	[-0.51, 0.02]	-1.78	0.08	-0.48	[-1.00, 0.05]	
AIC							99.10
BIC							109.90
R2							0.06
Sigma							0.50

hsa-miR-877-5p

Parameter	Coefficient	95% CI	t(59)	p	Std. Coef.	Std. Coef. 95% CI	Fit
(Intercept)	-4.24	[-5.73, -2.74]	-5.54	< .001	0.18	[-0.12, 0.47]	
AGE	0.00	[-0.01, 0.02]	0.57	0.57	0.07	[-0.18, 0.33]	
BMI	-0.01	[-0.04, 0.03]	-0.28	0.78	-0.04	[-0.29, 0.22]	
Group [IFG]	-0.37	[-0.72, -0.02]	-2.08	0.04	-0.55	[-1.08, -0.03]	
AIC							131.15
BIC							141.87
R2							0.08
Sigma							0.65

hsa-miR-152-3p

Parameter	Coefficient	95% CI	t(60)	p	Std. Coef.	Std. Coef. 95% CI	Fit
(Intercept)	-0.15	[-0.72, 0.42	-0.52	0.61	0.17	[-0.11, 0.46]	
AGE	0.00	[0.00, 0.01	1.53	0.13	0.19	[-0.05, 0.43]	
BMI	-0.01	[-0.02, 0.01	-0.72	0.47	-0.09	[-0.33, 0.15]	
Group [IFG]	-0.14	[-0.28, -0.01	-2.11	0.03	-0.55	[-1.06, -0.04]	
AIC							10.11
BIC							20.90
R2							0.12
Sigma							0.25

hsa-miR-93-5p

Parameter	Coefficient	95% CI	t(60)	p	Std. Coef.	Std. Coef. 95% CI	Fit
(Intercept)	2.33	[1.66, 3.00]	6.84	< .001	0.15	[-0.14, 0.44]	
AGE	0.00	[0.00, 0.01]	0.87	0.38	0.11	[-0.14, 0.36]	
BMI	0.00	[-0.01, 0.02]	0.42	0.67	0.05	[-0.20, 0.31]	
Group [IFG]	-0.14	[-0.30, 0.01]	-1.80	0.07	-0.48	[-1.01, 0.04]	
AIC							30.41
BIC							41.20
R2							0.07
Sigma							0.29

hsa-let-7i-5p

Parameter	Coefficient	95% CI	t(60)	p	Std. Coef.	Std. Coef. 95% CI	Fit
(Intercept)	3.30	[2.74, 3.86]	11.56	< .001	0.13	[-0.16, 0.43]	
AGE	0.00	[-0.01, 0.00]	-1.06	0.29	-0.14	[-0.39, 0.12]	
BMI	-0.01	[-0.02, 0.01]	-0.95	0.34	-0.12	[-0.37, 0.13]	
Group [IFG]	-0.11	[-0.24, 0.02]	-1.61	0.11	-0.43	[-0.96, 0.09]	
AIC							7.61
BIC							18.40
R2							0.06
Sigma							0.25

Supplementary Table S2: Transcription factor binding site prediction

miRNA	Transcription factor	Raw p-value	R	R-square	Transcription factor binding sites
let-7i-5p	Sp4	1.97E-04	0.88	0.86	1
let-7i-5p	Patz1	4.94E-04	0.84	0.82	8
let-7i-5p	E2f2	6.53E-04	0.83	0.80	7
let-7i-5p	Plagl2	1.42E-03	0.79	0.76	2
